# The outcomes and prognostic factors of patients requiring prolonged mechanical ventilation

**DOI:** 10.1038/srep28034

**Published:** 2016-06-14

**Authors:** Chih-Cheng Lai, Jiunn-Min Shieh, Shyh-Ren Chiang, Kuo-Hwa Chiang, Shih-Feng Weng, Chung-Han Ho, Kuei-Ling Tseng, Kuo-Chen Cheng

**Affiliations:** 1 Department of Intensive Care Medicine, Chi Mei Medical Center, Liouying, Taiwan; 2 Departments of Internal Medicine, Chi Mei Medical Center, Tainan, Taiwan; 3 Chia Nan University of Pharmacy & Science, Tainan, Taiwan; 4 Department of Healthcare Administration and Medical Informatics, Kaohsiung Medical University, Kaohsiung, Taiwan; 5 Medical Research, Chi Mei Medical Center, Tainan, Taiwan; 6 Department of Safety Health and Environmental Engineering, Chung Hwa University of Medical Technology, Tainan, Taiwan

## Abstract

The aims of this study were to investigate the outcomes of patients requiring prolonged mechanical ventilation (PMV) and to identify risk factors associated with its mortality rate. All patients admitted to the respiratory care centre (RCC) who required PMV (the use of MV ≥21 days) between January 2006 and December 2014 were enrolled. A total of 1,821 patients were identified; their mean age was 69.8 ± 14.2 years, and 521 patients (28.6%) were aged >80 years. Upon RCC admission, the APACHE II scores were 16.5 ± 6.3, and 1,311 (72.0%) patients had at least one comorbidity. Pulmonary infection was the most common diagnosis (n = 770, 42.3%). A total of 320 patients died during hospitalization, and the in-hospital mortality rate was 17.6%. A multivariate stepwise logistic regression analysis indicated that patients were more likely to die if they who were >80 years of age, had lower albumin levels (<2 g/dl) and higher APACHE II scores (≥15), required haemodialysis, or had a comorbidity. In conclusion, the in-hospital mortality for patients requiring PMV in our study was 17%, and mortality was associated with disease severity, hypoalbuminaemia, haemodialysis, and an older age.

In this era of technologically advanced medical care, mechanical ventilation (MV) has become one of the most frequent invasive devices applied to critically ill patients^1^. Although many patients may survive through the acute stage of critical illness, approximately 3–13% of mechanically ventilated patients experience difficulty weaning off MV and thus require prolonged mechanical ventilation (PMV; the use of MV for at least 21 days)^2^,^3^,^4^. Over the last decade, the number of patients requiring PMV has rapidly increased and was closely related to an increase in aging populations, patients with multiple comorbidities, and technological improvement^5^,^6^. Thereafter, several issues have been raised regarding this emerging population of patients requiring PMV, including a high consumption of healthcare resources and high medical expenses, difficulty in assessing outcomes and prognostic factors, and the time to introduce palliative care.

However, understanding the outcomes and prognostic factors of patients requiring PMV is important for physicians, patients, and their families, so good communications and a consensus on care plans can occur. In fact, a significant number of patients using PMV are likely to be near the end of life, and some may prefer palliative care to preserve quality of life, rather than prolong life expectancy with life-sustaining therapy (ie PMV). Despite several studies having investigated this critical issue, most were conducted in Western countries^2^,^7^,^8^,^9^,^10^,^11^,^12^,^13^,^14^,^15^,^16^,^17^,^18^,^19^,^20^. In Asia, only a few studies conducted in Korea and Taiwan ever reported the outcomes and prognostic factors of patients requiring PMV^21^,^22^,^23^,^24^,^25^. In Taiwan, the Bureau of National Health Insurance (NHI) of Taiwan, which is a mandatory universal health insurance program (since 1995) for the 23 million residents of Taiwan and covered up to 99% of the Taiwanese population by 2007, developed an Integrated Prospective Payment (IPP) program for patients requiring PMV to efficiently control the use of intensive care resources in 2000. In this system, MV care is divided into four types according to the duration of MV use: fee-for-service intensive care unit (ICU) (up to 21 days), respiratory care centres (RCC) (≤42 days), capitation respiratory care ward (RCW), and per-month home ventilator service^26^. The objective of RCC admission is to aggressively wean patients off PMV who have a stable hemodynamic status. In Taiwan, most studies were based on the National Health Research Institutes database; information regarding disease severity and real-time clinical or laboratory data was lacking^22^,^23^,^24^,^25^. In a report from a Korean group, only 136 patients requiring PMV were enrolled; all studies conducted in Asia failed to provide a comprehensive understanding of this issue^21^,^22^,^23^,^24^,^25^. Therefore, a large-scale investigation containing detailed information on patients in Asia is necessitated. The aims of this study were to investigate the outcomes of patients requiring PMV and to identify risk factors associated with their mortality rate in a single medical centre.

## Methods

### Patients and hospital setting

This study was conducted in Chi Mei Medical Center, a tertiary referral hospital that has 96 adult ICU beds and 16 beds in the RCC. In our hospital, the RCC was established in 2000, and a specialist in pulmonary and critical care medicine treats all RCC patients. The criteria for RCC admission included a stable haemodynamic status with no vasopressor requirement, no new development of complicated arrhythmia or signs of acute coronary artery syndrome, stable renal function, normal acid-base balance, controlled infection, and aged >17 years. In our RCC, the nurse-to-patient ratio was 1:4, and one respiratory therapist is on duty during each shift.

In this retrospective study, all patients requiring PMV (MV use ≥21 days) who were admitted to the RCC were enrolled between January 2006 and December 2014. For patients with repeated RCC admissions, we recorded and analysed only data from the last RCC admission. Data were collected on a routine basis, and the analysis was conducted retrospectively. The study was approved by the institutional review board of Chi Mei Medical Center, and informed consent was waived.

### Variable measurement

The medical records of all recruited patients were retrospectively reviewed, and the following information was collected: age, gender, ICU type prior to RCC admission, PMV causes, duration of MV use, Acute Physiology and Chronic Health Evaluation II (APACHE II) score upon RCC admission, comorbidities, laboratory examination results, and ICU and RCC hospitalization costs. The causes for PMV were defined by chest physicians, such as (1) lung infection, (2) decompensated heart failure, (3) neuromuscular diseases, (4) infection other than pneumonia, and (5) others, including chronic obstructive pulmonary disease, restrictive lung disease, and decompensated gastrointestinal or hepatobiliary diseases. As in a previous study, comorbidities were defined as congestive heart failure, coronary artery disease, chronic obstructive pulmonary disease, interstitial lung disease, pulmonary hypertension, end-stage renal disease, liver cirrhosis, diabetes mellitus, acute or chronic encephalopathy, cancer, and immunosuppressant usage^27^. The primary outcome was in-hospital mortality.

### Statistical analysis

Continuous variables are reported as mean and standard deviation, and categorical variables are presented as count and proportion. Further, differences in baseline characteristics and clinical variables between survival and mortality groups were evaluated using the Student’s t-test for continuous variables and Pearson chi-square tests for categorical variables. Baseline characteristics and clinical variables with P values of <0.05 were enrolled as candidates for constructing multivariate logistic regression models. For determining the final prediction model, a multivariate logistic regression was based on a stepwise model-selection procedure, in which all candidate variables were inserted until non-effects entered or effect removed from the backward elimination, to determine adjusted odds ratios with 95% confidence intervals (95% CI), and to examine the association between predictive variables and the mortality rate. SAS 9.4 for Windows (SAS Institute, Cary, NC, USA) was used for all analyses. Significance was set at a P of <0.05 (two-tailed).

## Results

### Demographic characteristics

During the 9-year period, a total of 1,821 patients requiring PMV were identified from 27,654 MV users, and the overall incidence of PMV among MV cases was 6.58%. Of all patients requiring PMV, the mean age was 69.8 ± 14.2 years. Patient ages ranged from 18 to 96 years, and 521 patients (28.6%) were aged >80 years. Most of the patients were male (n = 1,078, 59.2%). Most of the patients were transferred from a medical ICU to the RCC (n = 1,031, 56.6%), and others were transferred from a surgical ICU (790, 43.4%). The APACHE II scores upon RCC admission were 16.5 ± 6.3. Further, 1,308 (72.0%) patients had at least one comorbidity. Pulmonary infection was the most common diagnosis (n = 770, 42.3%), followed by a neuromuscular disease (n = 561, 30.8%), decompensated heart failure (n = 210, 11.5%), and infection other than pneumonia (n = 144, 7.9%). In addition, 279 patients (15.3%) required maintenance haemodialysis during RCC hospitalization. Of the patients who were not weaned, 1,459 patients had an artificial airway before death or being discharged. Among them, 1,116 patients had a tracheotomy (76.5%), and others (343 [23.5%]) had trans-laryngeal intubation. In this study, serum blood urea nitrogen (BUN), albumin, creatinine, phosphate, and haemoglobin levels were 39.9 ± 29.8 mg/dl, 2.7 ± 0.5 g/dl, 1.6 ± 1.6 mg/dl, 3.5 ± 2.1 mg/dl, and 10.0 ± 1.4 g/dl, respectively. The average costs during ICU and RCC admission were US$11,131 and US$7,045, respectively.

### Outcome analysis

**Figure 1:**
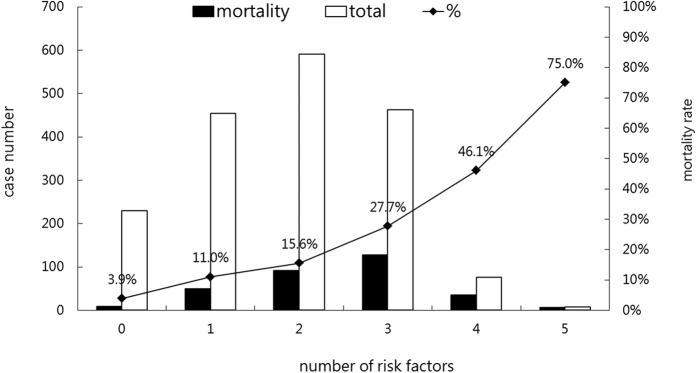
The number of cases and mortality rate for patients with risk factors.

A total of 320 patients died during this study, and the in-hospital mortality rate was 17.6%. We compared the clinical variables of patients with their survival and mortality outcomes (Table 1). Significant between-group differences were found with the following variables: age, ICU category, diagnosis upon RCC admission, comorbidity, APACHE II score, haemodialysis requirement, albumin, renal function, haemoglobin, MV duration, and hospital expenditure. Table 2 summarizes the risk factors associated with the in-hospital mortality rate determined using a stepwise logistic regression analysis. Patients who were aged >80 years had lower albumin levels or higher APACHE II scores, required haemodialysis, and had a comorbidity were more likely to die. Among the five risk factors, aged ≥80 years, albumin of ≤2.0 g/dl, APACHE II score of ≥15, haemodialysis requirement, and having a comorbidity were noted in 551; 178; 1,090; 279; and 1,308 patients, respectively. Figure 1 shows the number of cases and mortality rate for patients with one to five risk factors, and we found that patients with more risk factors had a higher mortality rate.

## Discussion

This study investigated a cohort of 1,821 patients requiring PMV, and the overall incidence of PMV for patients with acute respiratory failure requiring MV was 6.58% over 9 years. Several significant findings were observed. In this study, a total of 320 patients passed away during hospitalization, and the overall in-hospital mortality rate was 17.6%. In a multicentre study in the United States, Carson *et al*.^13^ enrolled 260 patients requiring PMV, and the in-hospital mortality rate was 28% in that study. In another multicentre study conducted in Brazil, Loss *et al*.^28^ studied 218 patients requiring PMV, and the hospital death rate was up to 65%. However, Damuth *et al*.^29^ conducted a meta-analysis and concluded that the outcomes of patients requiring PMV in a post-acute care hospital were worse in the United States than in other countries (in-hospital mortality rate was 31% [95% CI, 26–37] vs 18% [95% CI, 14–24], respectively). Therefore, outcomes for patients requiring PMV in Western countries varied according to different study settings and populations. In Taiwan, our study had a lower in-hospital mortality rate than that demonstrated in other countries. The difference may be because of the high selectivity of patients transferred from the ICU to the RCC.

In 2008, Carson *et al*.^12^ developed a scoring system and named it the Prolonged Ventilation score (ProVent score) to predict the 1-year mortality of patients requiring PMV. This system demonstrated its usefulness in medical care centres in five geographically diverse territories in the United States (in 2012) and in three primary ICUs within community hospitals in France (in 2014)^13^,^30^. For the ProVent score, factors included required haemodialysis, required vasopressor, thrombocytopenia, and aged ≥50 years. In Korea, Kim *et al*.^21^ discovered that thrombocytopenia and required vasopressor were significantly associated with 6-month mortality. In Taiwan, Lu *et al*.^25^ identified several prognostic factors including neoplasms, renal failure, shock, septicaemia, and non-alcoholic liver disease that were significantly associated with a lower survival rate in patients requiring PMV. In this study, several risk factors such as aged ≥80 years, APACHE II scores upon RCC admission of >15, albumin level of <2 g/dl, required haemodialysis, and had at least one comorbidity were related to mortality. In contrast to the ProVent scoring system and Korean study, our study did not find required a vasopressor or thrombocytopenia as outcome predictors in the model^4^,^12^,^13^,^21^,^30^. This discrepancy may have resulted because only patients with a stable hemodynamic status without vasopressor treatment could be admitted to the RCC. As for differences between this study and a study conducted by Lu *et al*.^25^, the study by Lu *et al*. was based on the National Health Insurance (NHI) system, governmental death registry data, and clinical data, but data on disease severity and laboratory examinations were lacking.

In the current study, all patients who had the five aforementioned risk factors died within 6 months. Our findings may help physicians predict clinical outcomes for patients requiring PMV. Further, once patients with the five aforementioned risk factors are identified, physicians should consider the possibility of a high failure rate with life-sustaining interventions and discuss the need for palliative care with patients and their families. Although only eight patients had these five risk factors, they may not be sensitive enough for overall outcome prediction. Consequently, palliative care plans for patients and their families should be encouraged for any patient on invasive MV, except for patients with multiple risk factors, and decision-making regarding palliative care or the withdrawal of life support should be rigorous and comprehensive. Therefore, in our study, the model provided useful information regarding outcome prediction and health care plans. However, additional large-scale studies are still warranted to validate our findings.

In this study, two factors were found to be associated with better outcomes. First, patients who were diagnosed with neuromuscular diseases when admitted to the RCC had a significantly higher survival rate than patients with other diagnoses. This is consistent with a study by Hung *et al*.^23^ that patients requiring PMV who had degenerative neurologic diseases or a stroke had a longer life expectancy than those in other subgroups. This subgroup may have fewer chronic cardiopulmonary diseases and thus may have a better opportunity for successful weaning or longer longevity. Second, patients who had a tracheostomy had better outcomes than patients with trans-laryngeal intubation. This finding in our study and a previous study indicate that a tracheostomy could lead to a higher survival rate in patients requiring PMV than patients who had trans-laryngeal intubation^31^. Hence, physicians should consider performing a tracheostomy for these patients.

Our study had two major limitations. First, only the in-hospital mortality rate was measured in the present study. We did not assess the outcomes of the patients after discharge, so long-term outcomes such as the 1-year survival rate are not indicated. In addition, we did not evaluate the patients’ quality of life or preference for PMV. Additional study is necessitated to clarify these issues. Second, our findings were based on a single institution. Therefore, generalizations from this study cannot be applied to patients in other hospitals or countries. However, our study enrolled a large number of patients over a long time period; thus, the results should remain representative of this specific population.

In conclusion, patients requiring PMV were selected based on strict RCC admission criteria, and their in-hospital mortality rate was <20%. Moreover, mortality was significantly associated with a high disease severity, hypoalbuminaemia, required haemodialysis, and an older age. In addition, the short-term mortality was 100% for patients with all five risk factors.

## Additional Information

**How to cite this article**: Lai, C.-C. *et al*. The outcomes and prognostic factors of patients requiring prolonged mechanical ventilation. *Sci. Rep.* **6**, 28034; doi: 10.1038/srep28034 (2016).

## Figures and Tables

**Table 1:** Comparison of clinical variables between survival and mortality outcomes.

Variables	Number (%) of patients with survival outcomes (*n* = 1501)	Number (%) of patients with mortality outcomes (*n* = 320)	P value
Age (years)	69.1 ± 14.5	72.8 ± 12.3	<0.001*
Age ≥ 80 years	399 (26.6)	112 (35.0)	0.003*
Gender			0.851
Male	887 (59.1)	191 (59.7)	
Female	614 (40.9)	129 (40.3)	
ICU category			<0.001*
Medical ICU	817 (54.4)	214 (66.9)	
Surgical ICU	684 (45.6)	106 (33.1)	
Diagnosis during RCC admission			<0.001*
Lung infection	620 (41.3)	150 (46.9)	0.071
Decompensated heart failure	165 (11.0)	45 (14.1)	0.123
Neuromuscular disease	504 (33.6)	57 (17.8)	<0.001*
Infection other than pneumonia	108 (7.2)	36 (11.3)	0.017*
Miscellaneous	104 (6.9)	32 (10.0)	0.061
Had at least one comorbidity			<0.001*
No	465 (31.0)	48 (15.0)	
Yes	1036 (69.0)	272 (85.0)	
APACHE II scores			<0.001*
<15	639 (42.8)	79 (25.0)	
≥15	853 (57.2)	237 (75.0)	
Laboratory examinations			
BUN (mg/dl)	36.0 ± 26.3	58.5 ± 37.6	<0.001*
Creatinine (mg/dl)	1.4 ± 1.4	2.3 ± 1.9	<0.001*
Haemoglobin (g/dl)	10.0 ± 1.4	9.6 ± 1.4	<0.001*
Phosphate (mg/dl)	3.5 ± 1.1	3.6 ± 1.5	0.428
Albumin (g/dl)			<0.001*
>2.0	1397 (93.1)	245 (76.6)	
≤2.0	103 (6.9)	75 (23.4)	
Required haemodialysis			<0.001*
Yes	187 (12.5)	92 (28.8)	
No	1314 (87.5)	228 (71.3)	
ICU cost (USD)	10599 ± 5839	13719 ± 9591	<0.001*
RCC cost (USD)	7207 ± 2804	6285 ± 3140	<0.001*
With or without tracheostomy^a^			<0.001*
Trans-laryngeal intubation	240 (20.4)	103 (36.3)	
Tracheostomy	935 (79.6)	181 (63.7)	

Data shown as number (%) or mean ± SD. Abbreviations: RCC, respiratory care centre; APACHE II, Acute Physiology and Chronic Health Evaluation II; ICU, intensive care unit; BUN, blood urea nitrogen; USD, United States Dollar.

^a^Of the patients who were not weaned, 1,459 patients had an artificial airway before death or being discharged.

^*^Statistically significant between groups (P < 0.05).

**Table 2:** Risk factors associated with in-hospital mortality determined with stepwise logistic regression.

Variable	Crude odds ratio (95% CI)	P value	Adjusted odds ratio (95% CI)	P value
Age				
<80 years	1.00		1.00	
≥80 years	1.49 (1.15–1.92)	0.002	1.47 (1.12–1.94)	0.006
Albumin				
>2.0 g/dl	1.00		1.00	
≤2.0 g/dl	4.15 (2.99–5.76)	<0.001	3.84 (2.73–5.38)	<0.001
APACHE II scores				
<15	1.00		1.00	
≥15	2.25 (1.71–2.96)	<0.001	1.63 (1.22–2.18)	0.001
Required haemodialysis				
No	1.00		1.00	
Yes	2.84 (2.13–3.78)	0.001	2.41 (1.77–3.29)	<0.001
Had at least one comorbidity				
No	1.00		1.00	
Yes	2.54 (1.84–3.52)	<0.001	1.98 (1.40–2.80)	<0.001
